# Developing a Solution for Mobility and Distribution Analysis Based on Bluetooth and Artificial Intelligence

**DOI:** 10.3390/s20247327

**Published:** 2020-12-20

**Authors:** Marius Minea, Cătălin Dumitrescu, Ilona Mădălina Costea, Ionuț Cosmin Chiva, Augustin Semenescu

**Affiliations:** 1 Department Telematics and Electronics for Transports, University “Politehnica” of Bucharest, 060042 Bucharest, Romania; ilona.costea@upb.ro (I.M.C.); ionut_cosmin.chiva@upb.ro (I.C.C.); 2 Department Engineering and Management for Transports, University “Politehnica” of Bucharest, 060042 Bucharest, Romania; augustin.semenescu@upb.ro

**Keywords:** Bluetooth sensors array, triangulation, received signal strength indicator, artificial intelligence, target tracking

## Abstract

The purpose of this research was to develop a simple, cost-effective, but enough efficient solution for locating, tracking and distribution analysis of people and/or vehicle flowing, based on non-intrusive Bluetooth sensing and selective filtering algorithms employing artificial intelligence components. The solution provides a tool for analyzing density of targets in a specific area, useful when checking contact proximities of a target along a route. The principle consists of the detection of mobile devices that use active Bluetooth connections, such as personal notebooks, smartphones, smartwatches, Bluetooth headphones, etc. to locate and track their movement in the dedicated area. For this purpose, a specific configuration of three BT sensors is used and RSSI levels compared, based on a combination of differential location estimates. The solution may also be suited for indoor localization where GPS signals are usually weak or missing; for example, in public places such as subway stations or trains, hospitals, airport terminals and so on. The applicability of this solution is estimated to be vast, ranging from travel and transport information services, route guidance, passenger flows tracking, and path recovery for persons suspected to have SARS-COV2 or other contagious viruses, serving epidemiologic enquiries. The specific configuration of Bluetooth detectors may be installed either in a fixed location, or in a public transport vehicle. A set of filters and algorithms for triangulation-based location of detected targets and movement tracking, based on artificial intelligence is employed. When applied in the public transport field, this setup can be also developed to extract additional information on traffic, such as private traffic flowing, or passenger movement patterns along the vehicle route, improved location in absence of GPS signals, etc. Field tests have been carried out for determining different aspects concerning indoor location accuracy, reliability, selection of targets and filtering. Results and possible applications are also presented in the final section of the paper.

## 1. Introduction

The research for this article was targeted in the following ways:

1. To develop a strongly cost-effective, but still efficient solution for intelligent detection of position and movement analysis of targets in a specific perimeter, using Bluetooth technology and artificial intelligence.
2. To discover, build and test the best configuration of BT sensors to be usable both on mobile platforms and outdoor/indoor infrastructures.
3. To evaluate the performances of the proposed setup and associated filtering algorithms in terms of location precision and possibility of tracking the movement of identified and tagged targets.
4. To evaluate the possibility of indoor locating and analyzing movements of persons in a dedicated area, support for route guidance and/or patients’ surveillance in hospitals.

These goals have been approached in different phases and experiments, of which one resulted in a patent proposal. In the first stages of the solution development, a series of field testing have been carried for determining different behavior of Bluetooth signals both in outdoor and indoor environments (a subway station). Secondly, different configurations for the specific Bluetooth sensors have been tested prior to decide over the most efficient and simple one. As a result of these experiments, the best configuration has been selected and a patent proposal has been registered. This paper presents the results of the third phase of field testing, along with filtering algorithms and future developments.

Compared to other, similar research, the proposed solution employs simple Bluetooth detectors and uses different techniques to improve location and tracking accuracy based on RSSI analysis, filtering and artificial intelligence. After locating a target or a set of targets with acceptable precision, the algorithms work together to filter them by successive positions and movement patterns. If used in a public transport vehicle (bus, tram, subway wagon, etc.), the proposed solution would serve to estimate the number of travelers in the vehicle, the travelers waiting in a station platform or even private traffic density/flowing next to the public transport vehicle. It could also serve as a detector for passenger flowing, improving the information regarding the level of service for the respective vehicle. When used in different, but connected infrastructure points, the solution could be employed for tagging a specific target and tracking its movements along the network. Applicability refers, but is not limited to information support for patients’ movements in hospitals, travelers in airport terminals or even persons carrying SARS-COV2, or other viruses’ recovery of direct contacts.

## 2. Related Work

There is a lot of research that refers to Bluetooth detection of people or vehicles carrying Bluetooth devices, with many approaches oriented towards usability in indoor locations, anonymous tracking of targets, or other similar applications. Bluetooth is a technology that is present in many portable devices such as smartphones, smartwatches, headphones, head units in cars, etc. It is a short-range communication technology via radio that allows low-cost, low-energy and low-bandwidth characteristics in many types of devices equipped with a Bluetooth module. Their diversity and number are increasing day by day. Therefore, we consider that, while still relatively imprecise, the solution of discovering and tracking such devices, supplemented with information regarding the representation among the total number of persons/vehicles carrying those devices represents a modern and cost-effective solution for gathering a lot of information on travel patterns, target density and field distribution, indoor location support or any other relevant data.

Signal strength information and timing is analyzed in [1] via simulation modeling with the purpose of setting an indoor corridor scenario. RSSI is used in Bluetooth technology to reduce the range of communication and therefore interferences between different devices in the neighboring areas, and to ensure a constant radio signal power. Timing is used by some researchers to determine distance to different APs, and in [1] it is stated that there are needed at least 2–3 inquiries in a single access point scenario to obtain usable results. Also, when employing this method for location, the solution is not very robust in relationship with APs’ locations (accuracy equals or is lower than 3 m in over 90% of the analyzed cases).

Another developed solution [2] propose a proximity estimation based on RSSI of Bluetooth signals and light sensing. This research explores the possibility of using Bluetooth as a face-to-face proximity estimation technique. The authors employ a propagation model considering transmission power, antenna gains, attenuation factor and distance. The conclusion of this study is that Bluetooth offers a usable solution for measuring face-to-face proximity.

Research regarding applicability of Bluetooth in patient tracking in a clinical environment is described in [3], where the authors investigate the feasibility of an indoor real-time location system based on Bluetooth and artificial intelligence. The study demonstrates the usability of Concurrent Neural Networks (CNN) and Artificial Neural Networks (ANN) in a combined solution with Bluetooth tagging for tracking persons in a dedicated network of sensors. Some solutions consider multiple neural networks architectures trained to deal with typical disadvantages of RSSI measurements, such as changes of RSSI values due to different orientations of the user’s receiver, presence of random obstacles in FOV, etc. The solution consisted of developing a predictive connection behavior oriented towards speeding the localization process, based on a NN architecture [4]. The main techniques for indoor localization are reviewed in [5], including RSSI measurement, AOA (Angle Of Arrival) or time analysis in a comparative study, along with the BER (Bit Error Rate), concluding that BER could be a good indicator between the communicating devices.

A specific triangulation method for the location estimation using Bluetooth signals is also presented in [6], where a solution to determine a more precise location considers the dependence between the distance and the received signal strength. As a definition, the RSSI provides an estimate between an optimal received signal strength (golden receiver power rank) and the actual received signal strength, not a direct value of the RF strength. The solution presented herein limits the possible usage only at distances of 8 m from an access point, combining a triangulation method with mathematical least square estimation. Compared to Wi-Fi 802.11 standards, where a beacon can be detected either in the proximity or few blocks away from the receiver, the Bluetooth technology has the advantage of low power RF signals used, that is the detection of a similar device is possible within meters (therefore, more accuracy for indoor localization, for example). Such an advantage is exploited by some authors [7], where a solution for detecting mobile BT devices is proposed based on deployment of a series of base stations in a defined territory and using mostly the same technology as in cellular networks. In several paperwork [8,9,10] an exploration has been performed on the possibility to determine location, based on the simple use of the signal strength as a proxy for length, with not very encouraging results. This is due mainly to propagation effects in a complicated environment that create many reflections, diffractions, absorption of signals and generating artifacts. The researchers state that by carefully controlling the power of transmitted signal, it is possible to obtain an accuracy of around 1.2 m [11]. The study on the feasibility of location determination based on cellular approach is continued in [12] with a specific combinatorial model for the cells and possible beacon locations, selected from candidate beacon positions. The authors declare that with this combinatorial model and beacons setup they were able to reduce the effects of propagation distortion in specific indoor environments. 

More recent research introduces an improvement to the triangulation method by using machine learning algorithms [13]. The setup includes a specific deployment of beacons along a path, that continuously transmit broadcast signals in a typical order. Data collection is performed by the mobile device, which also sends this information to a central server, where all computations are performed, and the result is finally sent back to the receiver. The solution also applies an average filtering for RSSI to improve accuracy. Location-based applications also find their place in advertising. Other research involves different procedures for improving the localization results [14,15,16,17,18]. The authors of [19] propose an optimized propagation model parameter for each Voronoi cell using the RSS and location coordinates of crowdsourcing points and estimate the RSS samples of interpolation points with the optimized propagation model parameters to establish a radio map—and reducing processing time. In [20] the authors present an algorithm named Dynamic Topology Detector (DTD) for extracting a GVD with topological information from a grid map. DTD further extracting connectivity among the GVD edges and vertices. DTD is claimed to provide efficient recovery mechanism to deal with local changes, working efficiently in dynamic environments. The study performed in [20] proposes employment of 3D Voronoi diagrams for the analysis and visualization of 3D points instead of the original data item. The proposed algorithm also computes the cluster of 3D points by applying a set of 3D Voronoi cells to describe and quantify 3D points. In [21] the authors consider the notion of structural complexity from a geometrical point of view and argue that it can be characterized using general metrics computed on three-dimensional sealed structural models. Similar approaches are described in references [22,23,24,25,26,27]. All these studies refer to localization and improving the discrimination between different radio-detected targets in the vicinity of the detection device(s). The solution provided in this paper also investigates the possibility of accuracy self-improvement, environmental mapping of fixed location detected devices, and multiple data collection along a route, if the detectors setup is installed, for example, on a public transport vehicle. The solution is based also on similar experimental results that have been previously investigated by the authors in close research activities—references [28,29,30].

## 3. Problem Definition

### 3.1. Framework

There are many situations where detection and tracking of specific vehicles, or persons is necessary. Starting with the public transport management systems, where assessment of traffic flowing is useful for dynamically allocating vehicles on route, and ending with public health domain, where analyzing previous contacts of an infected person helps reducing spread of an infectious disease, or virus, the possibility to discover, locate and track a person, or vehicle with a low-cost infrastructure and minimal energy consumption is very tempting. Also, especially for indoor environments, there are also situations where it is necessary to locate a person (e.g., traveler) and provide route guiding information, such as is the case for large airports terminals, subway multi-modal stations with many connections, and so on. As proven by many scientific and commercial papers, Bluetooth indoor positioning has a great market potential. Newer Bluetooth versions, starting with ver. 2.1, enabled usage of RSS (radio signal strength) information without extra time consuming. In location-based applications for radio transmissions, usually RX and RSSI are employed for measuring signal strength in the reception point. There is a difference between the two, namely that RX is measured in milliwatts [mW] or decibel-milliwatts [dBm], whereas RSSI is a signal strength percentage. A problem with RSSI that must be taken into account when using this method for location determination is that usually there is not a common relationship between a physical parameter and RSSI, as maximal values for RSSI could vary from 100 at some manufacturers to 127 to others. Moreover, the RX reference value might also differ from different producers. Therefore, we consider that it would be still possible to use RSSI readings for approximating location of a certain mobile device by analyzing several readings behavior in time and improving the trajectory traceability via artificial intelligence algorithms. Measurement accuracy might be improved if averaging techniques are also employed when possible. In previous research, we have performed several tests both in open field and indoor environments, trying to estimate the behavior of RSSI in different urban environments. We concluded that there are variations in the signal strength (based on RSSI observations) even if the two communicating devices are stationary.

Whatever application is intended to be used for, if RSSI is the parameter that is to be monitored to estimate distance, and to further calculate position of a mobile station with regard to a specific monitoring (fixed) station, a proper model for signal propagation would be always useful. Some authors [19] recommend certain models, based on experiments performed to determine the best suitable behavior for signal attenuation. One of these is denoted by:

(1) $$RSSI=-\left(10\;nlog_{10}d+AE\right),$$

(2) RSSI=−(10nlgd+AE)

where *d* is the distance between the communicating devices, *n* represents the signal transmission constant, *AE* is defined as the absolute energy—A comparison factor in terms of energy, taken at 1 m distance from the measuring point. Because the chosen configuration consists in three reference points (with fixed distances between them), which are evaluating the signal produced by a mobile station in the coverage area, we can consider that for a single pair of any two reference points the *n* factor can be evaluated as:

(3) $$n_{xy}=\left(\frac{RSSI-AE}{10lgd_{x-y}}\right)$$

where, for a more precise estimation, *AE* could be the average of three consecutive values of RSSI at 1 m from the measurement point:

(4) $$n_{xy}^{avg}=\left(\frac{RSSI-(\sum_{i=1}^{3}AE_{i})/3}{10lgd_{x-y}}\right)$$

with *A* continuously updating as time passes and measurements take place.

**Figure 1:**
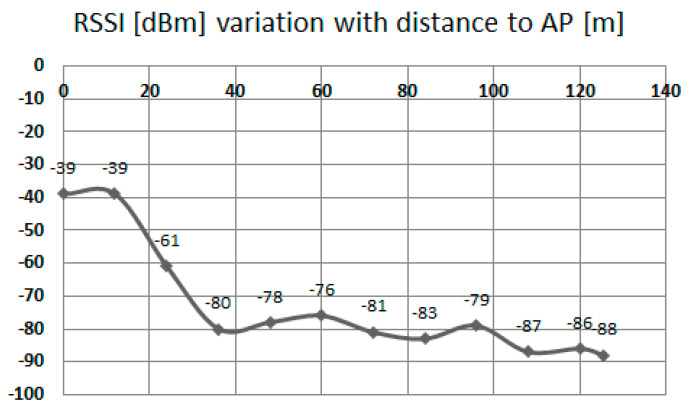
Outdoor variation of RSSI with distance, open field (from previous work, source: [17]).

RSSI also does not vary linearly with distance—Figure 1. Moreover, if the purpose of the application is to be used in public transportation traceability of travelers, propagation conditions may vary significantly. Therefore, the purpose of this application is not to precisely pinpoint a specific mobile target, but instead to obtain additional information sets from this technique, namely: analysis of density of mobile targets in a specific designated area, trajectory analysis of a mobile station, memorization of a specific configuration of fixed stations for improving location accuracy in public transportation etc.

The most frequently used methods for detecting a location of a mobile station, considering a set of three fixed beacons, are: time information, difference in time, AOA (Angle of Arrival), and signal strength. Each method may prove its advantages and drawbacks, ranging from the commercial availability of devices capable of delivering the required information, to the precision of the method itself. While the purpose of the developed application is to determine densities of mobile stations and trajectories of selected ones, instead of precise positioning, there is still a need for an appropriate model for the radio signals propagation. There are several scientific papers proposing different propagation models [14,15,16], but there will always be a difference compared to the real signal received, due to many random factors that occur in the real environment propagation. However, it is beneficial to explore the available models to determine which is more appropriate in a given environment, as this might help adjusting the results obtained when employing AI algorithms. Therefore, we proposed ourselves to employ this method more for movement traceability and density analysis than for exactly locating a certain mobile device.

To determine the position of a MAC address, two methods are employed: RSSI only-based trilateration and triangulation.

**Figure 2:**
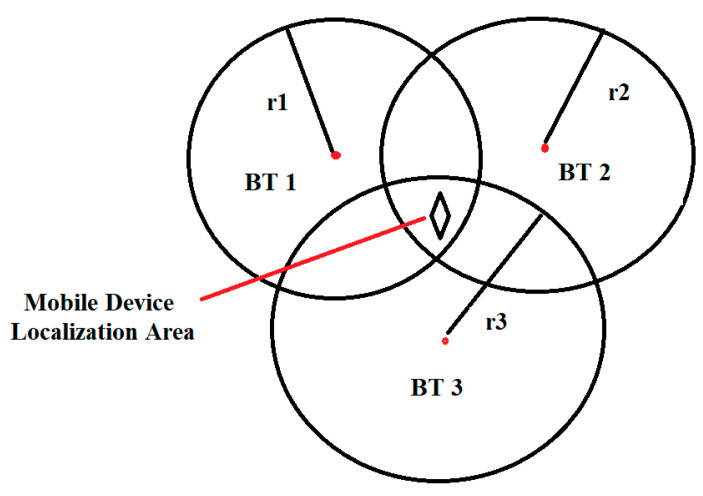
Mobile device location area using RSSI-based trilateration method.

Bluetooth trilateration uses signal strength (RSSI) to estimate the user’s distance from each of the three installed transmitters. The method we implement is spherical trilateration/triangulation with Voronoi and Delaunay algorithms, using the power of the reception signal (RSSI), the MAC address and the real physical coordinates of the installed Bluetooth access points. Based on the RSSI received by the Bluetooth sensor from the mobile terminal, the distance between the access points and the mobile terminal can be determined. To achieve triangulation, we need at least three sensors arranged in the area of interest. From the tests we found that the power of the received signal decreases exponentially depending on the distance between the transmitter, receiver and the noise factor in the environment. In conclusion, this dependence can be used in determining the distance of reception and mobility. The estimated distance for the receiving cell depending on the signal strength can be approximated by a circle whose radius is around the access point (reception). Thus, the intersection of the three radii related to the access points determines a point or area where the mobile terminal is located. The presented model can be defined by the following system of equations:

(5) $$d_{1}^{2}={\left(x-x_{1}\right)}^{2}+{\left(y-y_{1}\right)}^{2}$$

(6) $$d_{2}^{2}={\left(x-x_{2}\right)}^{2}+{\left(y-y_{2}\right)}^{2}$$

(7) $$d_{3}^{2}={\left(x-x_{3}\right)}^{2}+{\left(y-y_{3}\right)}^{2}$$

where *x*_1_, *x*_2_, *x*_3_, *y*_1_, *y*_2_, *y*_3_ are the coordinates of the three Bluetooth access points and *d*_1_, *d*_2_, *d*_3_ represents the estimated distances. Figure 2 shows the solution of distances equations which determine the points of intersection of the circles that determine the triangulation cells thus creating an interior location area.

Thus, it is possible to determine the location using RSSI as a segment between two values. We calculate the observation error using the formula:

(8) $$\Delta =\sqrt{\left(\sigma \cdot t\right)+A^{2}}$$

where Δ is the observation error expressed in dBm, *σ* is the standard deviation divided by the square root of the number of measurements, and *A* is the observation error of the mobile device.

**Figure 3:**
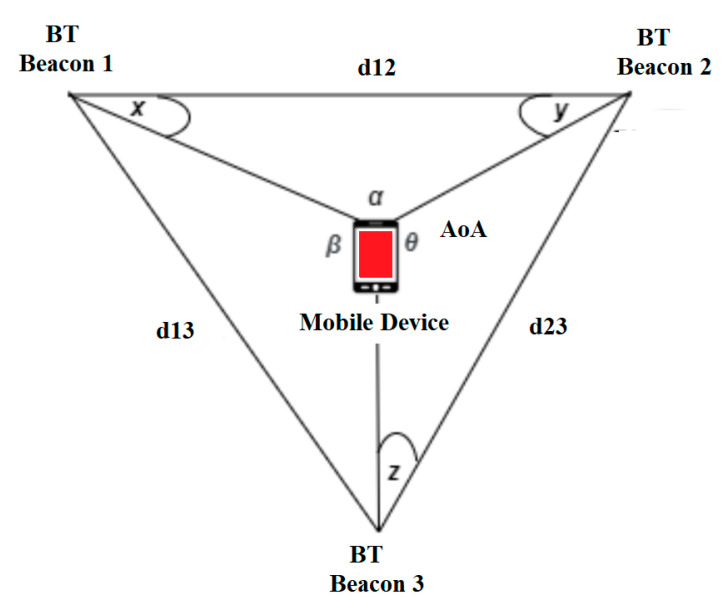
Triangulation location estimation using AoA, distance and time information.

To increase the location accuracy, we will also use the triangulation method based on AoA, distances and time information. Triangulation is a technique for calculating the position of a point that is based on a known distance between two or three reference points and the angles measured using the feature of finding the Bluetooth direction between those reference points up to that point, for example AoA. Unlike trilateral, which implements only distance measurements, the triangulation technique uses angle measurements. Using this method, we may calculate the location of any point in 2-D, considering the three angles between the point and three other reference points, subsequently implementing the Artificial Intelligence algorithm. Figure 3 shows the triangulation method, where: d_12_, d_23_ and d_13_ are the distances between the beacons BT 1–2, BT 2–3 and BT 1–3. The angles x, y and z represent the known angles between the mobile terminal and the BT 1, 2 and 3 beacons. Using these known measurements, the triangulation technique allows us to calculate the angles α, β and θ. Finally, we get the location of the mobile device. Due to AoA the triangulation provides a more precise location of the mobile device compared to the trilateral technique.

### 3.2. Preliminary Work

As the method chosen for detecting and analyzing traceability, as well as density of mobile targets is based on Bluetooth anonymous surveillance, it has been considered most appropriate to begin with some field testing on the Bluetooth signal propagation behavior in specific, controllable conditions. In order to keep the lowest cost for the future development of the proposed solution, the analysis focused on the behavior of RSSI in different conditions, for studying the extent of variations in both stationary and moving positions of the mobile (target) station, at different distances from the fixed reference measuring points. The initial phase of tests (not described in this paper) has begun in an outdoor open field, by determining evolution of RSSI readings according to distance from the transmitter. These tests were also part from another research, aimed to discover the best configuration of sensors in a setup of three devices—thought so for later performing triangulation based on triple pairs of stations. 

## 4. Field Test Experiments

### 4.1. Test Bed Setup

For setting up the indoor testing conditions, a laboratory in the University Politehnica of Bucharest has been prepared, by marking around measurement positions (MP_ij_) separated 1 m each other and placing close to the center of the room three measurement stations (MeS_1_–MeS_3_). The configuration chosen for these measurement stations has been previously analyzed and proposed in a Patent (mentioned at the end of this paperwork), with the purpose to be used in a public transportation vehicle, for determining several parameters, among which: An estimation of the number of travelers carried, estimation of the travelers waiting in stations, and private traffic vehicles flowing. This is not subject of the current paper, but because some results were obtained along with experience from this previous process, we have employed the same configuration. In the laboratory, the interior of the room where tests have been performed is separated by a simple plasterboard wall with a window, containing a metallic structural frame.

**Figure 4:**
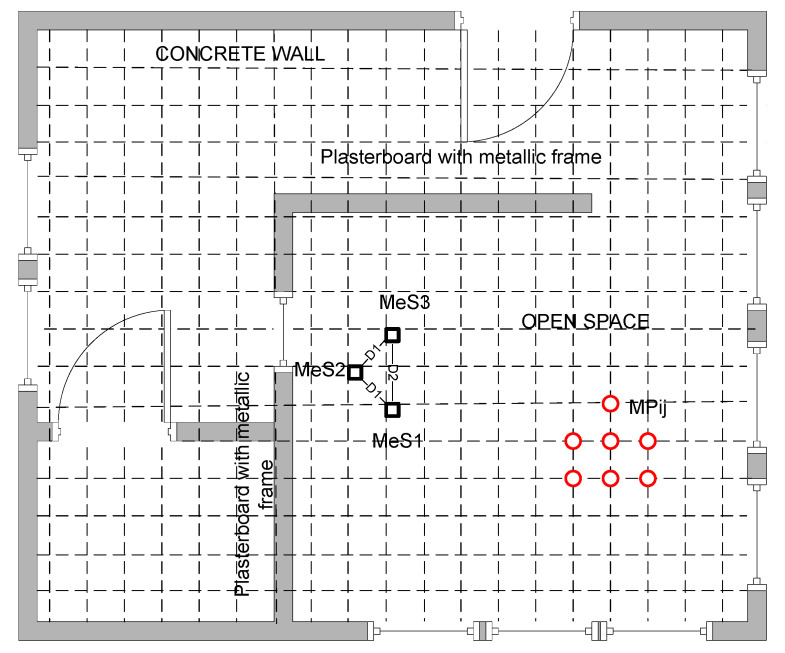
Laboratory setup for measuring variation of RSSI according to distance from MeSs and over time.

**Figure 5:**
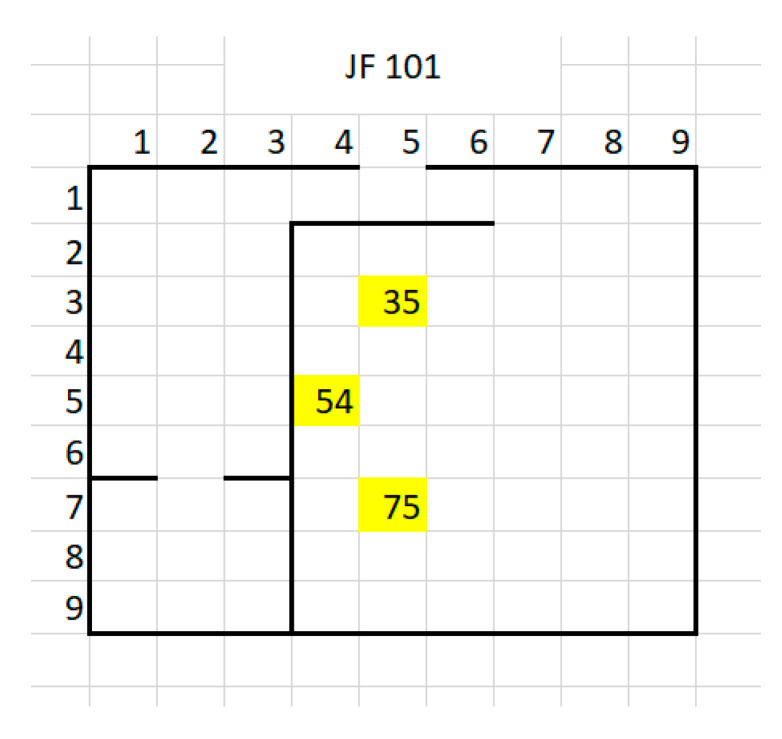
A more schematic representation of the positions where measurement stations were placed (75, 54 and 35 are the marked positions of MeS_1_ to MeS_3_).

The entire inner space was measured, and position points have been marked for each intersection between two dotted lines in Figure 4, separated by 1 m distance. Thus, a total of 99 points have been considered for evaluation. For each MeS and each measurement point MP_ij_, a set of three different RSSI readings has been performed. This was established in such a way to determine variations in RSSI readings in stationary conditions (where only channel usage by other devices might influence the analysis of the received RSSI values). No moving objects were interposed between the communicating points during the whole period of testing. Equipment employed: Nokia 7.1 mobile phone with Android One and Bluetooth Scanner software in MeSs and Oppo AX7 CPH 1903, with Android 8.1.0 and Color OS v5.2 as MP_ij_ tracked mobile station. In Figure 4, the distances MeS_1_-MeS_2_ and MeS_2_-MeS_3_ are D_1_ = 2236 m, and MeS_1_-MeS_3_ is D_2_ = 3 m. For the averaged set of measurements regarding each evaluation point, a map with variation of RSSI was produced. A more schematic setup of the measurement stations positions is presented in Figure 5 below.

### 4.2. Results of the First Indoor Testing Phase

As stated in beginning of this paper, the RSSI reading is not a perfect solution for determining location and distance between different Bluetooth communicating devices. But the purpose for which this solution was developed is to determine traceability of a mobile target, density of targets in a designated area, or proximity contacts. Therefore, the post-detection processes that take place are extremely important: they need to extract, filter, and produce an analysis result of the successive detection points for a specific target. This is the main reason why an accurate location technique is not necessary for each point of detection, because several points are analyzed in a successive matter, to determine the path of the target and its neighbors along it. However, it will always be important to study the behavior of RSSI in stationary conditions, in order to introduce possible filtering, or interpolation techniques for improving the location accuracy. The results of this first phase of the field tests are presented in Table 1, for average values for measuring the MeS_1_ distance.

**Figure 6:**
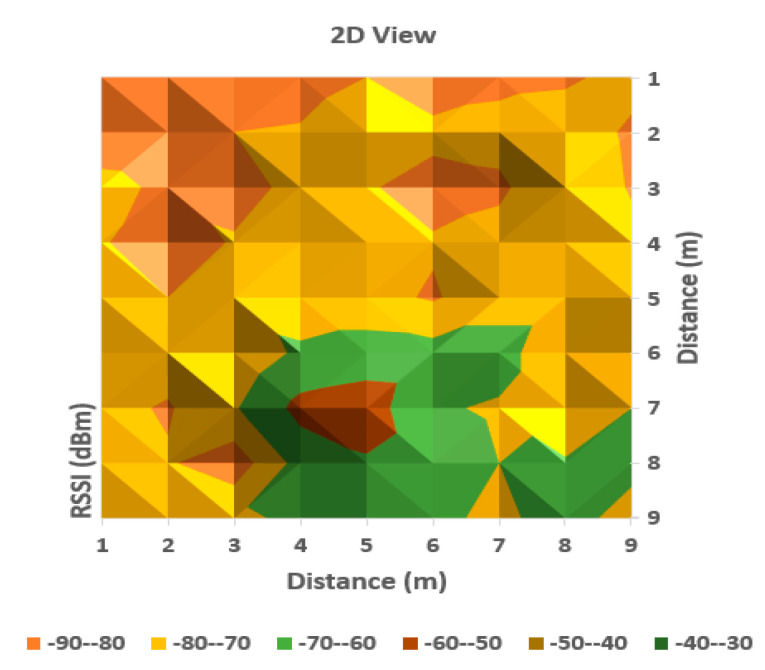
A two-dimensional representation of signal variation with different coloring according to RSSI measured values (Measurement point: MeS_1_).

**Figure 7:**
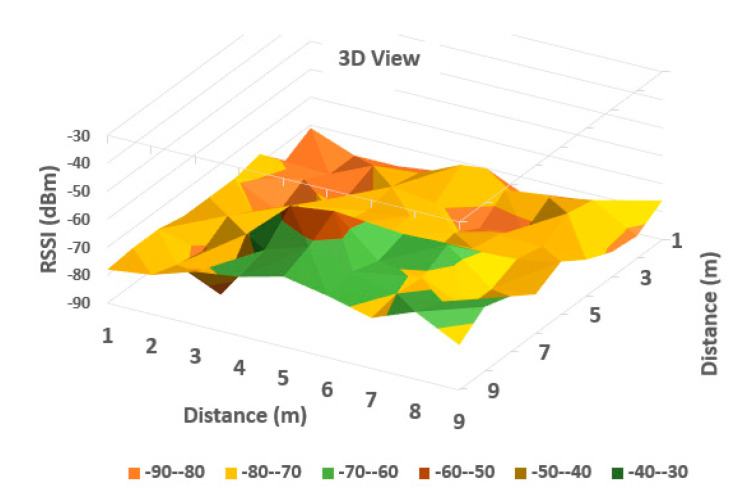
A three-dimensional representation of signal variation with different coloring according to RSSI measured values (Measurement point: MeS_1_).

It can be observed from Table 1 that the averaged read values of RSSI vary between neighboring cells in a certain amount, but in general, the loss of signal power can be observed according to distance between the communicating devices. For a more obvious highlighting of how the signal behaves, a 2D representation is presented in Figure 6 and Figure 7 below—histogram with different colors allocated to RSSI levels (color codification shown in the bottom of the image).

A more suggestive representation of the evolution of the signal levels is shown in a three-dimensional histogram, shown in Figure 4. Peaks represent high values or level, while valleys are low reception values of RSSI.

**Figure 8:**
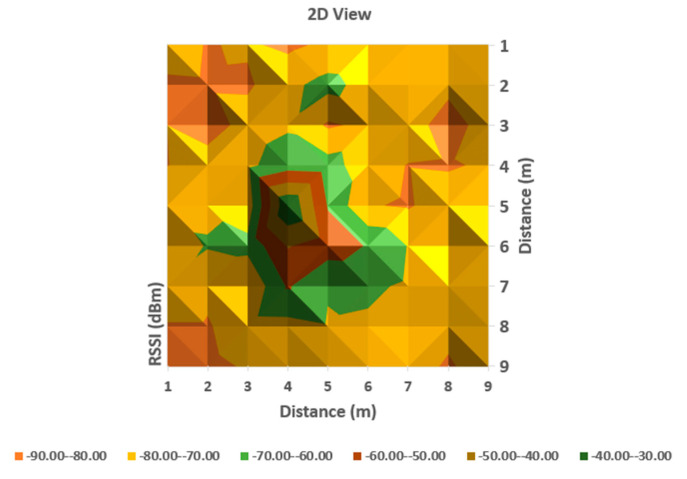
A two-dimensional representation of signal variation with different coloring according to RSSI measured values (Measurement point: MeS_2_).

**Figure 9:**
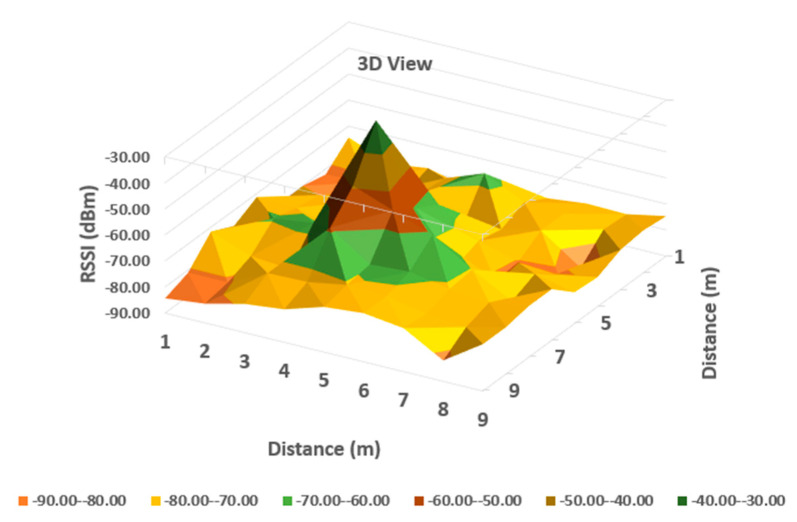
A three-dimensional representation of signal variation with different coloring according to RSSI measured values (Measurement point: MeS_2_).

**Figure 10:**
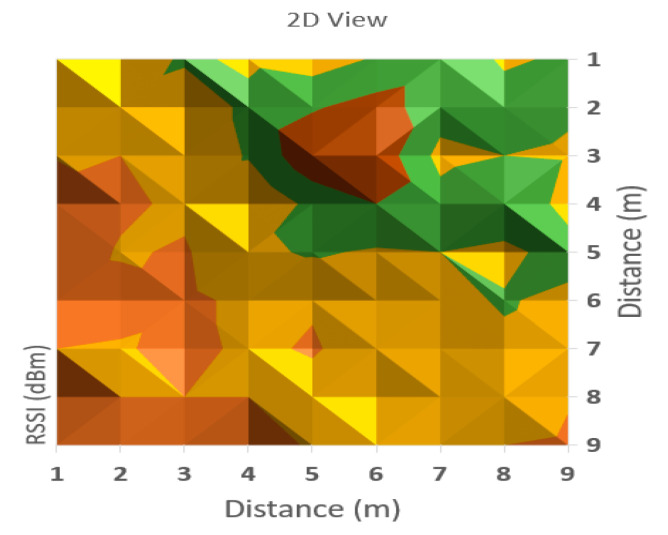
A two-dimensional representation of signal variation with different coloring according to RSSI measured values (Measurement point: MeS_3_).

**Figure 11:**
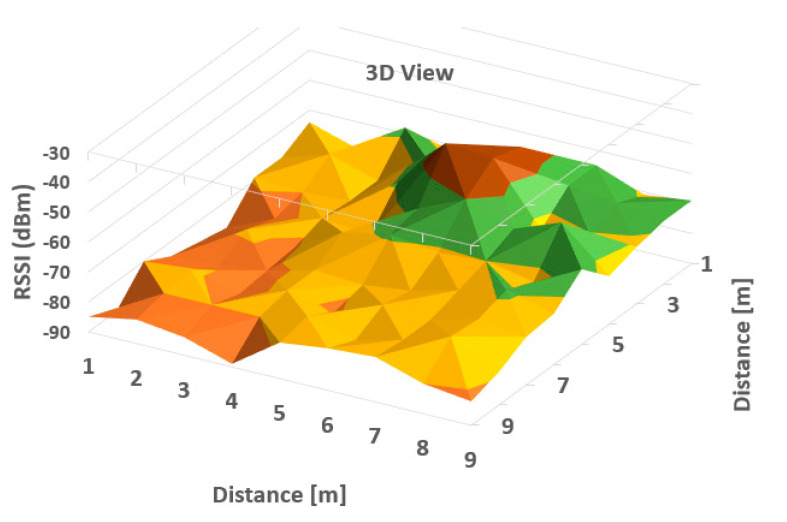
A three-dimensional representation of signal variation with different coloring according to RSSI measured values (Measurement point: MeS_3_).

As can be observed in Figure 6 and Figure 7, the behavior of RSSI is in general with respect to distance to transmitter, but there are variations in some directions, and even in the same position. As the histograms presented in these figures represent in fact values averaged for each point (from a set of three independent readings each), there still remains important variations in recorded levels. Therefore, it is considered that simple evaluation of RSSI is not sufficient for an accurate localization of a target, even if improved with differential procedures for triangulation. Specific filtering algorithms and comparison with previous recorded positions, to build a trajectory for the target are thus important consecutive actions. In Figure 6 and Figure 7, the peak levels are recorded in the vicinity of the measuring point (transmitter is in close vicinity with the measurement station), with some variations (green color around the peak colored in brown). This behavior is also observed for each from the other observing positions (Figure 8, Figure 9, Figure 10 and Figure 11 below). Table 2 shows the average measurement values for the MeS_2_ distance.

Table 3 shows the average measurement values for the MeS_3_ distance.

### 4.3. Discussion Regarding the First Test Phase

The first part of the field tests aimed to initially evaluate the variations of RSSI determined in quasi-stationary conditions. During this phase, no persons or objects were interposed between the measuring stations and the mobile device, placed alternatively in each of the 99 positions spread in the laboratory. All variations in signal strength are due either to equipment and software processes, or to variations of channel usage by other, neighboring Bluetooth devices. It can be observed from Figure 7, Figure 9 and Figure 11 with three-dimensional representations, that are most suggestive, that while the signal strength generally conforms to the free-space attenuation pattern, there are some isolated islands where strength experiences some increase. Vertical “views” presented in Figure 6, Figure 8 and Figure 10 give an idea on the pattern of sensitivity of the receiving stations, on the one hand, and suggest that this pattern is not quite perfectly circular. Therefore, one factor that should be taken into consideration when deploying the system on a wider scale would be to adjust the filters and algorithms to keep as circular as possible the sensitivity of the receiving station (antenna sensitivity to horizontal position of the received target). Also, the horizontal sensitivity of the reception position might be influenced by the relative vicinity with concrete walls and metallic frames. The electromagnetic field decreases with distance (according to the model presented in Section 3.1), but there is a random variation in the RSSI reading, that may have as main causes:

- load of the communication channels (the location where the tests have been performed was in a laboratory in a university, where access points and other Bluetooth devices were present in the vicinity)
- natural behavior of the e.m. field in the building environment
- Bluetooth RSSI evaluation process (timing of sampling, antenna pattern of the mobile stations etc.); however, the measuring device was not moved during the tests.

### 4.4. Second Test Phase—Influence of Interposing/Moving Obstacles on the RSSI Readings

In order to determine the influence in RSSI readings that any interposing object may have, a typical situation has been taken into consideration: influence of the human bodies. This situation is frequently met especially in public areas where people move in different directions and the absorption of electromagnetic waves may also influence the values of RSSI. A second set of tests was performed in the laboratory, in the same conditions described above, but with successive interposing of human bodies between the transmitter and the receiver.

Below are presented results of these tests.

(a) Influence of the receiver antenna orientation—vertical angle: as in the normal application, the device that will be employed for RSSI measurement will be a GSM terminal, it has been considered to determine if the orientation of this component according to horizontal plane may have influence on the RSSI readings.

**Figure 12:**
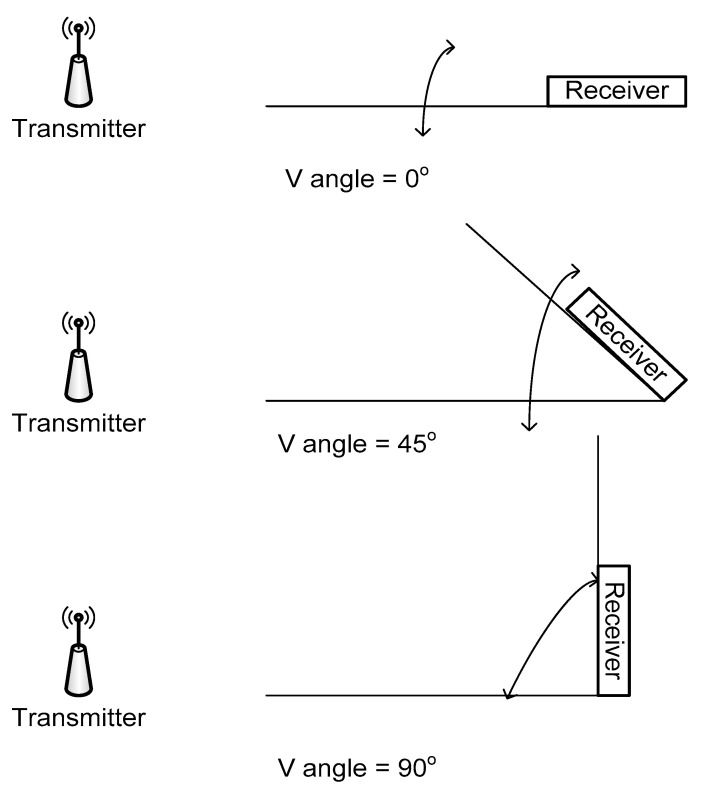
Angles of receiving station according to horizontal plane.

**Figure 13:**
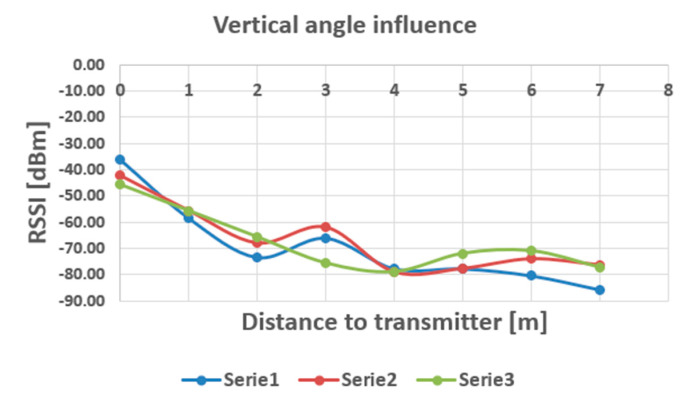
Influence of vertical position on the RSSI readings (Serie 1—V = 0^o^, Serie 2—V = 45^o^, Serie 3—V = 90^o^).

The measuring station has been then positioned in three angles according to the horizontal plane, receiver and transmitter both placed horizontally (V angle = 0°), at 45 degrees (V angle = 45°), and at 90 degrees (V angle = 90°), as shown in Figure 12 and Figure 13 shows how the RSSI influences the arrangement of the reception sensor in a vertical position.

Influence of the receiver antenna orientation—to determine if the orientation of this component according to direction to transmitter may influence the RSSI readings.

**Figure 14:**
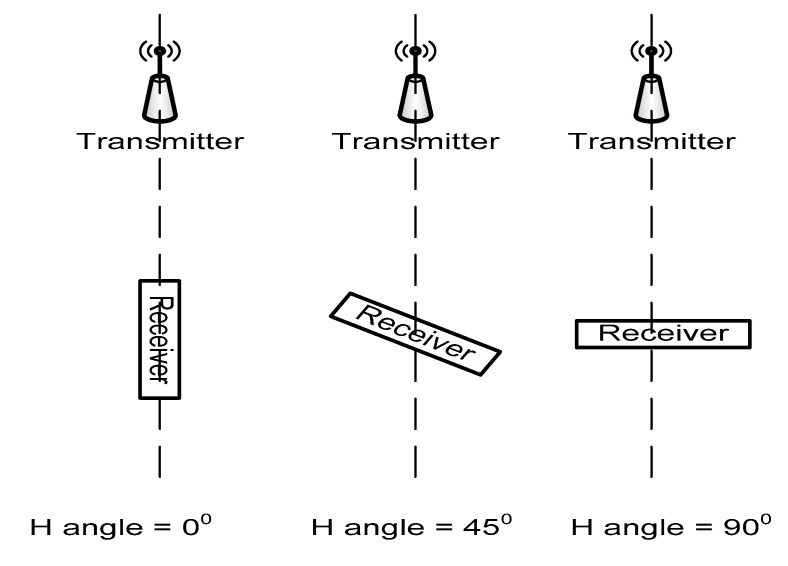
Angles of receiving station according to horizontal plane.

(b) The setting of the measurement conditions is presented in Figure 14.

**Figure 15:**
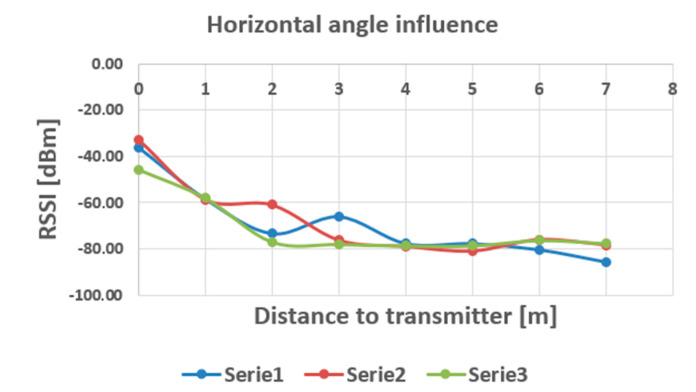
Influence of horizontal position on the RSSI readings (Serie 1—H = 0^o^, Serie 2—H = 45^o^, Serie 3—H = 90^o^).

The influence of this position is presented in Figure 15.

**Figure 16:**
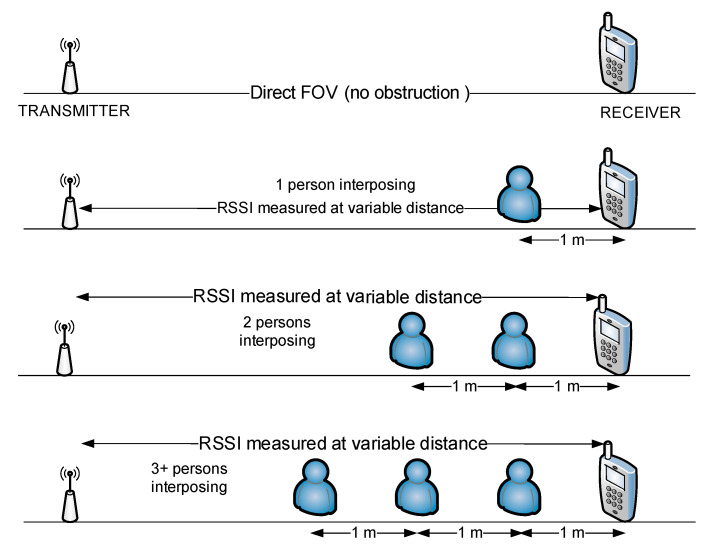
Successive positioning of interposing human bodies between the transmitter and the receiver, FOV line.

In the third part of this experiment, we placed the two communicating devices in straight position (Field of View) and we started to interpose one by one, persons in the direct line of communication, continuing the determination of RSSI values in these variable conditions. The overall arrangement for this phase of experiments is shown in Figure 16.

**Figure 17:**
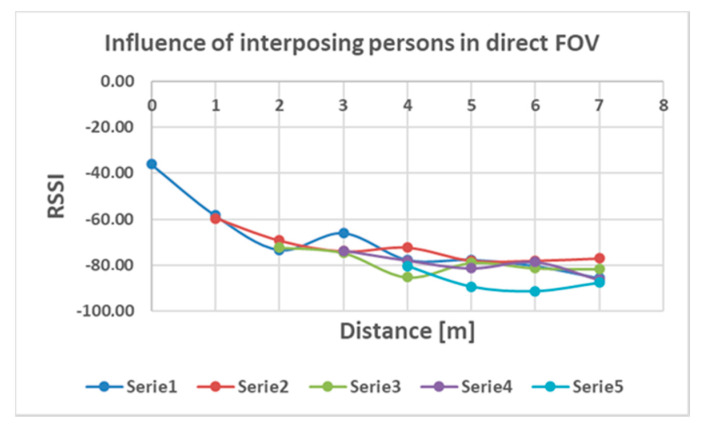
Readings of RSSI for variable number of persons sitting in direct communication line (Serie 1—no persons (FOV), Serie 2—1 person, Serie 3—2 persons, Serie 4—3 persons, Serie 5—4 persons).

The influence of the human bodies on the RSSI readings is presented in Figure 17.

### 4.5. Discussion on the Second Test Phase and Preliminary Conclusions

According to the results obtained in these laboratory tests, it seems that the RSSI readings are rapidly dropping in the vicinity of the transmitter, then the variation is slowing down up to distances larger than 8 m and it appears the field keeps in a relative slow variation around −80 dBm. However, still in constant environment conditions (urban scenario, with several Wi-Fi access points in the surroundings and random Bluetooth devices passing by), the RSSI is experiencing variations ranging between 0 and 12–14 dBm, which gives a poor precision in terms of distance measurement based on RSSI levels. If only a single measuring station would be employed for such kind of applications, then the precision would be very poor.

When we varied the vertical angle of the measuring station, the influence between different angles appears to be negligible, ranging approximately in the same domain of variation of the stationary field in the measurements with fixed station orientation. As it can be noticed in the diagrams shown in Figure 13 and Figure 15, the most remarkable variation can be observed in the interval between 2 to 4 m (approximately) distance from the transmitter, where dynamics as large as 18 dBm have been recorded especially when the horizontal position of the receiving station was altered. Beyond 4 m, the variations are at the most comparable with the variation of the stationary field, which is a good thing for trajectory, or targets density analysis, when these targets are situated further from the measuring points.

Regarding the influence of human bodies in the direct line of transmission, the results are presented in Figure 17. In this case, it appears that the number of interposing persons between the two communicating devices has a more constant influence on the whole range of distance measured, RSSI readings varying around 10 dBm. Therefore, we can consider that in case of denser zones, with more people, precision of localization and trajectory monitoring may decrease. In these series of tests, only the receiving station’s position has been altered to determine influence on the RSSI levels recorded. No transmitter position was modified. However, in the real world, mobile detectable devices are various (handsets, smartwatches, BT headphones etc.) and held in different positions. Therefore, a specific random variation will probably be recorded due to these factors, also decreasing the precision of localization.

It can be once more concluded that, based simply on RSSI readings, a precision localization would not be obtained, except, maybe ideal conditions with less influence from other mobile stations (free e.m. environment) and with steady positions of the transmitter and receiver. Therefore we consider very important that in the first phase, the RSSI data collection should be performed not by one but by several measuring stations (in this paper a configuration of three is proposed), and triangulation procedures should then be applied from each pair of measuring stations, finally comparing results. This more complicated methodology should improve the localization precision. Then, in the second phase, a deep analysis of results, based on artificial intelligence should be employed for determining a more accurate position for targets, trajectory estimation (including tracking) and targets’ density evaluation (including estimative minimal distance between targets).

## 5. Post-Processing of Data—Artificial Intelligence Algorithms

**Figure 18:**
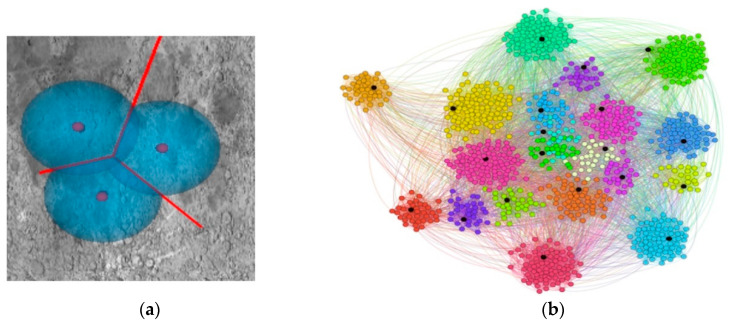
Voronoi diagrams. (**a**) Illustration of Voronoi partitioning in 2D Euclidean space for 3 BT cells. (**b**) Voronoi diagrams for graphs represented by the k-mean application developed by us. The generating nodes are displayed in black.

To introduce the clustering method, we first take a look at the Voronoi partitioning method in 2D Euclidean space. Consider a set of points in a 2D plane, distributed to locally form groups. The local density of the points inside the territories is computed and then select the generating points of the Voronoi cells inside their territory with radius r, as seen in Figure 18. Each point in the plane is assigned to a Voronoi cell with the nearest generating point. As a result, the points are divided into groups by Voronoi cells. To apply the previous theory to networks, we need to transform the graph into a metric space. First, we calculate a value of the distance between each two nodes, which is the length of the shortest route between them. The length of any route is equal to the sum of the link lengths along the route, where we defined the link length as the inverse of the link grouping coefficient (ECC). The ECC of a link between node *i* and node *j* is calculated as:

(9) $$C_{i,j}=\frac{z_{i.j}+1}{min\left[\left(k_{i}-1\right),\left(k_{j}-1\right)\right]}$$

where *k*_i_; *k_j_* are the link numbers of the nodes, *z_i,j_* is the number of triangles to which the link belongs and *min[…]* is the number of potential triangles to which it could belong, because this is less than the link numbers of those two adjacent nodes, minus one (link examined). If the value 1/*C*_*i,j*_ is high, then the link probably connects nodes from different clusters.

Once the distance has been determined, the next step is to establish the generating points. During the calculations a selection method of Voronoi generating points based on the relative local density of the nodes has been used. It works on a subgraph formed by the first neighbors of node *i*. For this subgraph, it is determined:

(10) $$\rho_{i}=\frac{m}{m+k}$$

where *m* is the number of links inside the neighborhood and *k* is the number of links coming out of the subgraph. If we determined the generating points as a function of the distance *1/C_i,j_* and the radius *r*, then we obtained a clustered graph. However, the best strategy for Voronoi partitioning is to increase the *r* value and examine the quality of the clustering, for example by cluster modularity.

Unsupervised learning or clustering does not require the presence of the training set with a priori pre-classified data. The purpose of unsupervised learning is defined by the need to explore data for the discovery of intrinsic structures. The user explores the data to find new, interesting, and useful structures. Among the most important methods of unsupervised learning is clustering, which organizes data into similar groups called clusters in such a way that the instances in one group are similar in some respects and totally different from the data in the other groups. The quality of different clustering methods is differentiated by using objective functions. The quality of clustering refers primarily to homogeneity within groups and separability between the groups resulting from the clustering process. The unsupervised learning process is an iterative one. The most widely used partition algorithm is the k-means algorithm, which has become the exponent of an entire category of algorithms. The popularity is given by the simplicity of the implementation, scalability, efficiency, and speed of convergence.

**Figure 19:**
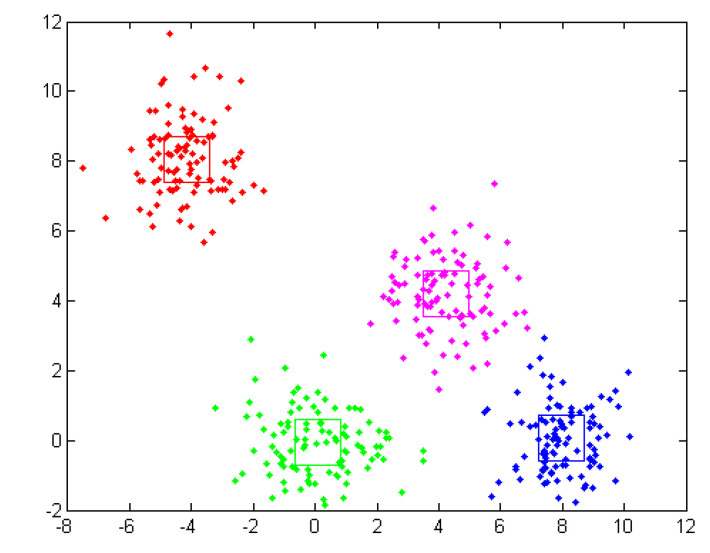
Graphic representation of some clusters and highlighting of centroid.

The clustering process results in one or more groups representing the spatial positioning of the data characteristics. Within a cluster, the points are closer together in relation to a common center than in relation to the centers of other clusters, as shown in Figure 19.

K-means is one of the simplest algorithms in unsupervised learning, with good results obtained by solving well-known clustering problems. In the classical procedure, the data set is to be divided into *K* pre-set groups. The main idea is to define *K* centroids, one for each cluster. In this case, the placement of centroid in the search space is crucial, as placing it in different locations can lead to different results. Looking at the initial dataset as a group of entities, we will have the entities, along with the corresponding feature vectors that will be classified into the *k* different groups. An entity *i*, $i=\bar{1,n}$ will be assigned to a group *C_j_,* $j=\bar{1,k}$ with the centroid *K_j_* being closest to this vector, and *K_j_* is updated after each assignment, using the formula: $K_{j}=K_{j}+\frac{1}{nr_{j}}\left(z_{i}-K_{j}\right)$, where *nr_j_* represents the number of entities belonging to the group *C_j_* (cluster size). In the classical problem in which K-means are used, the *k* centroids are initialized either with randomly generated values, from the definition domain of the problem, or with *k* from the entities of the data set, also chosen at random.

The algorithm can be considered:

- choose the Initial Centroid (*Z*, *k*)
- how long (there are still differences between current and previous centroid)
- assign entities to clusters
- recalculates centroid
- how long

**Figure 20:**
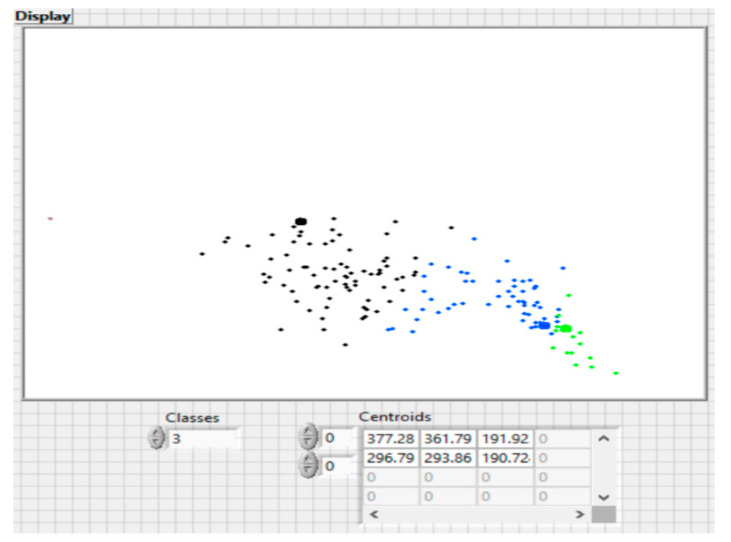
The results of the proposed k-mean algorithm for 3 classes.

The results of the algorithm are presented in Figure 20.

The purpose of this algorithm is to minimize the objective function, a quadratic error function. The objective function is given by the expression:

(11) $$J=\overset{k}{\underset{j=1}{\sum }}\overset{n}{\underset{i=1}{\sum }}{\left\|z_{i}^{\left(j\right)}-K_{j}\right\|}^{2}$$

where $\left\|z_{i}^{\left(j\right)}-K_{j}\right\|$ is the distance measured between the point $x_{i}^{\left(j\right)}$ and the centroid *K* of the cluster *C_j_*.

## 6. Results of Post-Processing

The experiments were performed in a dedicated laboratory at the Politehnica University of Bucharest. The architecture of the space and the location of the BT sensors 1, 2 and 3 were presented in Figure 4. The red dots in Figure 4 represent the test points. Also, subjects walked through all three rooms (Figure 4). RSSI values were made over a time and used for positioning.

Table 4 shows the actual distance (m) and RSSI values from BT beacons 1, 2 and 3. Table 5 shows the values for the actual distance, position and accuracy calculated by the system and the detection probability.

**Figure 21:**
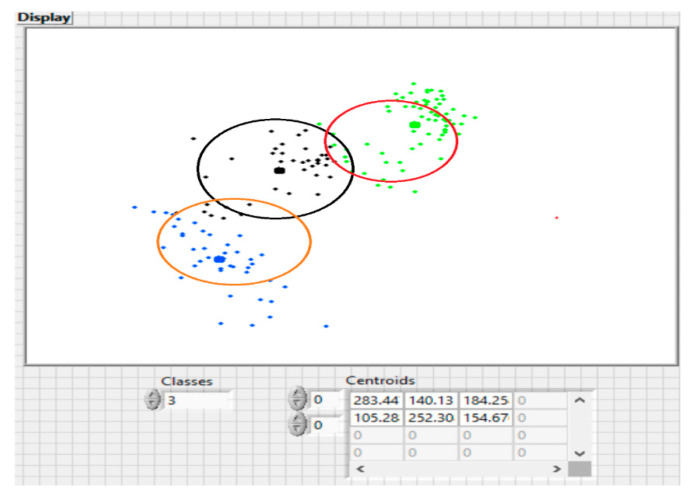
Detecting RSSI by classes using the proposed algorithm (red—2 m; blue—12 m; green—6 m).

The results shown in Figure 21 demonstrate the accuracy of positioning the subjects relative to Bluetooth beacons using triangulation. The accuracy of the Bluetooth-based reception system can be improved if more access points (BTs) configured in a cellular architecture are used. The propagation of radio waves from the indoor environment (walls, glass and metal objects) represent a considerable obstacle in making position determinations using trilateration or triangulation.

The BT trilateral method usually offers a low location accuracy, which is the reason we have chosen to use the triangular method with Voronoi type cell maps. To improve the prediction models for localization, the following methods can be used: increasing the number of BT detection beacons and installing them in a cellular architecture, making location maps based on fingerprints based on advanced RSSI measurements, building radio maps and creating a database using MAC addresses and related RSSI values.

Using the database with MAC and RSSI addresses (Big Data), together with Voronoi and Clustering algorithms, we can determine the tracking of topics and determine the route it has taken. Target tracking is the action of predicting the future trajectory of the subject depending on previous positions. The accuracy of the predictions depends on the accuracy of detecting the past and present positions of the subject. Unfortunately, due to the parasitic echoes generated by the objects inside, the limitations of the sensors (number and technical parameters) and the position of the subjects can be determined approximately based on the accuracy of the proposed calculation algorithms.

The measurements can highlight various random features and can be constrained by different initial conditions that depend on AoA and the relative position of the BT beacon. In addition, the fact that a subject can accelerate, can execute rapid changes of direction, thus deviating from a movement (trajectory) with constant speed, makes tracking a subject with path prediction a difficult task.

To *estimate the trajectory*, we use the concept of mapping and simultaneous location. This concept of simultaneous mapping and location is a technique created by the robotics community to analyze the possibility of an autonomous vehicle to start from an unknown location and assimilating information about the environment in which it operates, creates a map of active space that is built incrementally. By using BT reception sensors, RSSI, MAC data (fixed and mobile) and simultaneously filling-in new data in the generated map, the pedestrian/vehicle can be located. A practical solution regarding location and mapping is an invaluable value in terms of achieving an intelligent concept for determining human mobility but also urban, for Intelligent Transportation System (ITS) applications.

In what concerns the evolution of the topic of mapping and simultaneous location, a series of concepts and approaches were developed. It is possible to classify the different approaches according to the method of representation used to make the map, which is a key point, as it determines the type of information explicitly expressed in the model. The most common methods of representation are:

- Occupancy grids. They describe the surroundings by dividing the space into several regular cells and assigning a probabilistic value that establishes empty, unknown and occupied areas.
- Topological maps. An example of such maps is the Voronoi diagrams.
- Maps based on particular features. The basic elements of the representation of the environment are achieved by using a set of geometric primitives, such as points, lines and planes previously extracted from raw sensor data.
- Maps made from successive scans. The raw data of the sensors are aligned directly through a process of translation and rotation by finding a maximum overlap with the data contained in the previous scan. This results in map segments that are merged with each new data acquisition. It is suitable for use in situations where no simple geometric representation of the environment can be obtained.

In this article we propose a trajectory estimation algorithm based on topological maps (computational geometry) developed on Voronoi diagrams and combined with the Delaunay triangulation algorithm with applicability on the cellular system developed based on receptive BT cells. This algorithm can also be used to detect urban mobility of vehicles and pedestrians. In computational geometry many problems involve the decomposition of a geometric shape into several figures. One way to do this is to find the components behind this figure by overlapping the objects found over the main figure. These found objects stand at the base of the main geometric shape. One of these methods, quite well known, is the triangularization that divides the inner regions of the geometric shape into triangles. In most cases the values associated with the coordinates of the vertices can determine if one triangulation is better than another. The best method is to find an optimal triangulation for a given problem and to generate all possible triangulations by comparing them with each other. This is feasible for a polygon with several vertices. But as these peaks increase, the number of triangularizations increases. As we know there are several methods of triangulating a set of given points, but sometimes we need a triangularization with certain properties.

**Figure 22:**
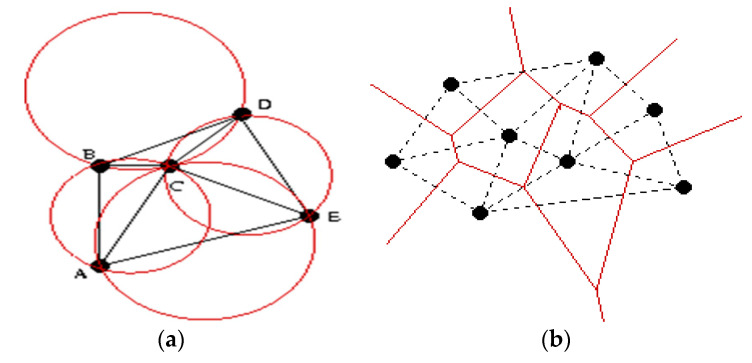
Representations of the Delaunay triangulation (**a**), Voronoi diagrams combined with the Delaunay algorithm (**b**).

One of the best known is the so-called Delaunay triangulation. The Delaunay triangulation of a given set of points is defined by the following property: AB is an edge (side) of a Delaunay triangularization, if there is a circle through which the edge AB passes so that any other points outside the circle joined by a point C, which is not equal to A or B, forms with this point a triangulation. Equivalently, all triangulations for a set of points will have circumscribed circles (Figure 22).

**Figure 23:**
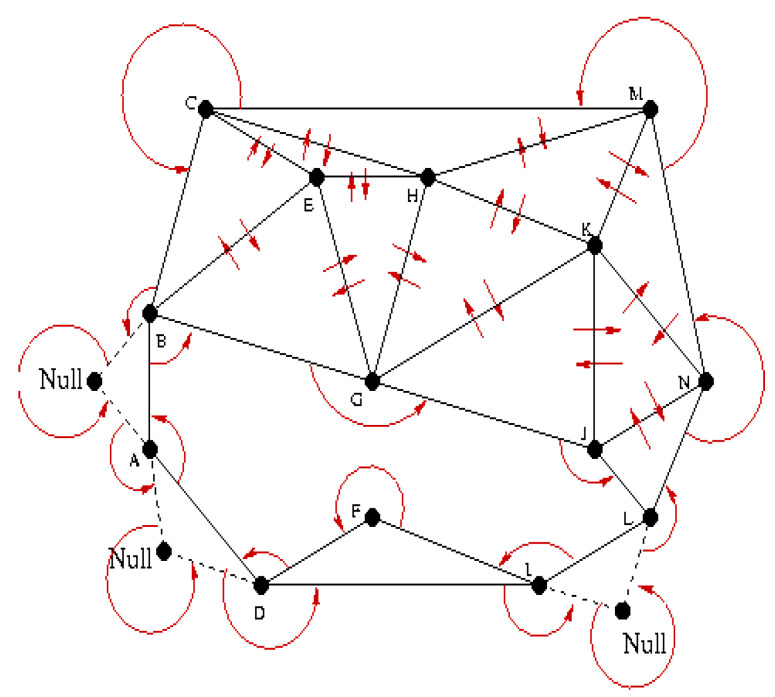
Representation of the implementation of the Delaunay algorithm for triangulation.

The implementation steps of the Delaunay algorithm: the triangle is defined by 6 member data, 3 vertices and 3 adjacent triangles. If a triangle is divided, 2 vertices remain, and we will make the 3rd NULL. Each triangle is generated such that the vertex one is smaller than the vertex 3, and the second vertex is oriented clockwise with respect to the vertex 1 and the vertex 3 with respect to 2. If there is no adjacent triangle opposite a vertex, then the neighboring triangle is assigned to be the first triangle encountered in the path of the respective peak. The process is repeated until the structure is completed and the triangularization is completed. The implementation of the algorithm is presented in Figure 23.

**Figure 24:**
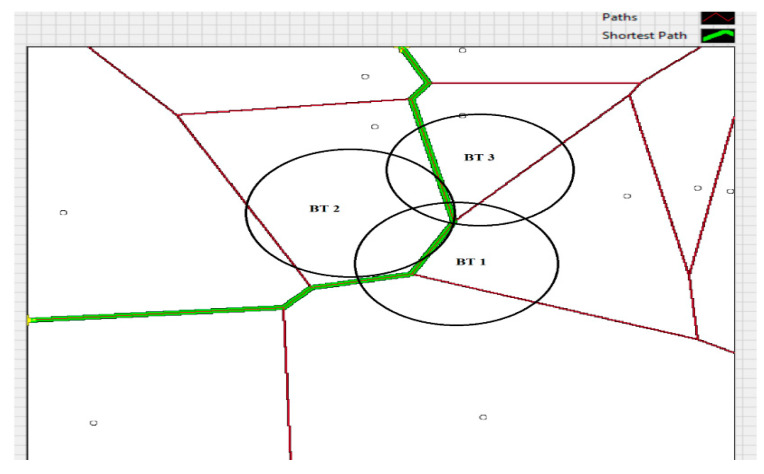
Tracking trajectory for subject 5, using the proposed system.

Figure 24 shows the application developed for estimating the travel trajectory, based on the topological map with the Voronoi diagram for calculating the shortest path based on the Delaunay triangulation algorithm. The calculation of the travel trajectory is based on the BT 1–3 reception sensors and the values obtained for RSSI and AoA related to a MAC address.

As a second issue, for the detection of human/urban mobility, we consider a stochastic version of the trajectory detection method described above. The essence of the new method consists of the random selection of the generating points and the use of the Voronoi cohesion matrix. Each node has the possibility of being a generating point. For a given selection of generating nodes, the Voronoi cohesion matrix is calculated, which is the probability of co-locating a pair of nodes, indicating the probability of intra-community and inter-community pairs. In the considered form, the values are higher for intra-community pairs than for inter-community pairs. Following this method, a clustering is obtained via stochastic steps. Knowing that each node can be randomly selected as a generating point, the steps of the algorithm are the following:

- randomly choose several generating nodes and perform a graph—Voronoi partitioning on the distance along the shortest route. Then, for each pair of nodes, determine their co-location.
- repeat this partitioning several times and calculate the Voronoi cohesion matrix, which is defined as the average of the co-location matrices. These cohesion matrices are represented so that the nodes are ordered according to the “ground truth” information.
- we identified that the intra-modular and inter-modular nodes are somehow separate. Starting from this, an iterative change of the network topology is applied. A small percentage of low cohesion bonds are moved between unconnected nodes with high associated cohesion. As a result of the change, the structure of the community is preserved, but the separation between them is more clearly defined.

The algorithm has been tested on benchmarks. A benchmark has been generated with several levels, with 10 and 8 communities on the first and second level, respectively. We evaluated the cohesion matrix and the topological relocation at each cycle. As a result, 7 communities have been obtained in both cases (with and without topological relocation), but the cohesion matrix indicates a better clustering if we use topological ordering.

Before testing real-world networks, a brief look at how the Voronoi cohesion matrix is built is needed. An element of the matrix is a probability for the co-location of a pair of nodes, i.e., the probability that the two nodes belong to the same Voronoi cell. The matrix contains diagonal and non-diagonal probabilities. Following the sorting method according to the “ground truth” information, the nodes belonging to the same cluster are located with higher probability on the diagonal of the matrix. If necessary, when the cohesion matrix does not clearly show the communities, we use a method of increasing the contrast, during which we topologically move the weak links and then separate the communities by a threshold value. During the contrast enhancement method, communities can be determined as follows: all nodes receive a separate community tag, and in a loop, across all nodes, the community tag of the current node is assigned to those nodes that have not changed still the label and whose cohesion with the current node exceeds a certain threshold. Clustering real-world networks remains difficult. During community detection, the simple stochastic graph-Voronoi method generates the cohesion map, where the difference between inter and intra pairs is not significant.

In their light, the simple stochastic graph-Voronoi method works well on benchmarks. In the case of real-world networks, the contrast enhancement technique is also required, as well as the Delaunay triangulation algorithm. In some extreme cases, the combined method is also required, for example the use of the Kalman filter with fuzzy logic together with the Voronoi diagram and the Delaunay triangulation.

The Kalman filter consists of a system of mathematical equations based on minimizing the squares of the errors and thus represents an optimal estimator for position correction and estimation of the travel trajectory. The arguments supporting its use in the automation industry relate to:

- efficiency in implementation
- estimating past, current and possible future states
- measuring “hidden” states
- qualitative measurement of a prediction by variances
- robustness; the filter handles very well in inaccurate models and is stable under specific conditions

The operation of the models is based on the following cyclic scheme: the first phase includes the temporary update in which an estimate of the state vector for the next step is made. In the next phase, an estimate of the state of the first phase is made by adapting it to a current measurement, so that it can be corrected. The adjustment difference is estimable, so that a prediction with the Kalman filter would reduce this difference between the theoretical and the real curve to a minimum, the system becoming more accurate. The mathematical model of the Kalman filter and the fuzzy logic for correcting the position of a vehicle, using the positioning data with the help of landmarks are described in the models we developed in [28,29,30].

## 7. Conclusions and Future Work

The purpose of this research was to develop a simple, energy-saving and environmental-friendly solution for locating, tracking and analyzing populations of Bluetooth targets, with different fields of applications, starting with the public transportation and ending with medical and epidemiologic enquiries.

The positioning accuracy of Bluetooth targets in indoor environments depends on the efficiency of the data post-processing algorithms. The activities presented in this paper focused mainly on determining indoor propagation conditions of BT radio signals and improving the current positioning algorithms (trilateral and triangular). An improved BT positioning algorithm is proposed by merging computerized geometry algorithms (Voronoi) and the k-mean cluster algorithm. The proposed algorithm is based on the location fingerprinting by making triangulation cells such as those used in robotics to determine the direction of movement. Using the acquired data, we also proposed a new fuzzy algorithm for estimating the trajectory of the subjects, thus resulting in a more precise location. Based on the proposed algorithms, in addition to positioning accuracy, we considered the following development directions: automatic creation of the database of fingerprints (maps) by automatically generating them using evolutionary algorithms to determine population mobility (e.g., Game of Life algorithm) and the possibility of automatically increasing the number of decomposition classes in the cluster algorithm by introducing a SOFM (Self Organizing Feature Map) neural network. Furthermore, the technique of automatically mapping the surroundings may also be used in future improvement of location accuracy of connected vehicles, creating a more accurate tactical image to the on-board unit and merging this information with the one collected by other sensors. Other applications, such as avoidance of car collisions with pedestrians can also benefit from this solution. We intend to perform more research in this direction.

## 8. Patents

Part of this research has been previously tested for developing a method for the anonymous collection of travelers flowing in a public transport system—and resulted in a Patent proposal: RO A/00493, “Method and System for Anonymous Collection of Information regarding Position and Mobility in Public Transportation, employing Bluetooth and Artificial Intelligence” in 2019.

## Figures and Tables

**Table 1:** Measured RSSI for MeS_1_ (position 75: row 7, column 5).

		Mobile Station (Column) Position								
Mobile Station (Row Pos.)		1	2	3	4	5	6	7	8	9
	1	−81.00	−87.67	−87.33	−85.33	−80.00	−87.00	−83.00	−82.00	−76.67
	2	−86.33	−87.33	−80.33	−79.33	−72.33	−77.33	−76.67	−73.33	−82.33
	3	−77.33	−82.33	−84.33	−77.00	−79.33	−84.00	−82.00	−71.33	−81.67
	4	−79.00	−87.33	−79.33	−79.00	−77.33	−79.33	−76.00	−76.33	−77.67
	5	−79.67	−80.67	−74.67	−77.33	−77.33	−81.67	−74.33	−74.67	−71.67
	6	−79.33	−75.67	−78.33	−68.00	−65.00	−66.33	−66.00	−78.33	−76.67
	7	−77.00	−81.00	−71.00	−57.33	−55.33	−69.00	−71.00	−78.33	−70.33
	8	−77.00	−79.00	−86.00	−66.33	−61.00	−66.00	−70.67	−69.33	−67.67
	9	−78.33	−76.00	−71.33	−69.33	−65.00	−68.33	−72.67	−66.67	−74.33

In Table 1, A = 19.67, *n* = 5.64 so RSSI=−(10·5.64lgd+57.66) for the considered values, relatively to MeS_2_.

**Table 2:** Measured RSSI for MeS_2_ (position 54: row 5, column 4).

		Mobile Station (Column) Position								
Mobile Station (Row Pos.)		1	2	3	4	5	6	7	8	9
	1	−74.33	−80.67	−78.67	−82.33	−78.00	−79.00	−77.33	−77.67	−74.67
	2	−82.00	−81.33	−81.67	−72.33	−66.67	−74.00	−74.67	−77.00	−73.67
	3	−80.00	−86.67	−75.00	−71.33	−81.33	−78.00	−74.67	−85.33	−74.67
	4	−80.33	−73.33	−72.00	−64.33	−65.67	−76.00	−80.33	−81.00	−78.67
	5	−71.33	−71.67	−75.67	−30.67	−60.67	−79.33	−81.00	−74.67	−78.00
	6	−76.00	−69.33	−67.33	−49.33	−55.00	−60.67	−70.33	−78.67	−70.33
	7	−71.67	−77.33	−78.67	−59.00	−69.33	−68.33	−71.33	−72.67	−70.33
	8	−81.00	−81.00	−73.67	−73.33	−70.67	−71.33	−74.33	−75.67	−72.00
	9	−84.33	-82.67	−79.00	−77.33	−72.67	−71.33	−73.33	−82.00	−72.00

**Table 3:** Measured RSSI for MeS_3_ (position 35: row 3, column 5).

		Mobile Station (Column) Position								
		1	2	3	4	5	6	7	8	9
Mobile Station (Row Pos.)	1	−74.00	−79.67	−68.00	−72.33	−75.00	−69.33	−65.00	−71.00	−69.00
	2	−79.33	−74.33	−79.00	−67.00	−61.33	−56.33	−65.00	−67.00	−69.33
	3	−79.67	−80.67	−79.00	−69.33	−50.00	−53.67	−73.67	−70.67	−71.67
	4	−89.67	−82.33	−78.33	−80.00	−67.33	−60.67	−66.67	−60.00	−74.67
	5	−87.33	−79.00	−81.33	−72.00	−69.00	−71.67	−70.67	−73.67	−65.67
	6	−85.33	−84.33	−82.00	−78.00	−79.67	−77.33	−73.67	−69.33	−73.00
	7	−80.67	−79.33	−83.33	−78.33	−81.33	−75.67	−76.33	−72.67	−74.33
	8	−91.00	−80.67	−80.67	−80.33	−76.67	−80.67	−76.33	−71.67	−79.33
	9	−85.33	−82.33	−84.33	−89.00	−78.33	−76.00	−75.67	−80.00	−82.00

**Table 4:** RSSI levels for BT 1–3.

Subjects	Distance (m)	BT1/RSSI (dBm)	BT2/RSSI (dBm)	BT3/RSSI (dBm)
S 1	1	−33.3	−138.8	−43
S 2	5	−51.8	−75.1	−62.5
S 3	10	−62.9	−76	−78
S 4	12	−72.7	−88.2	−86.02
S 5	15	−76.7	−92.1	−82.05

**Table 5:** Error observation vs distance for BT 1–3.

Subjects	Distance (m)	Average Detection Probability Observation Error (%) (m)	
S 1	1	0.45	98
S 2	5	0.98	97
S 3	10	1.05	92
S 4	12	1.25	92
S 5	15	1.66	90
